# Differences in Transcriptional Activity of Human Papillomavirus Type 6 Molecular Variants in Recurrent Respiratory Papillomatosis

**DOI:** 10.1371/journal.pone.0132325

**Published:** 2015-07-07

**Authors:** Caroline Measso do Bonfim, João Simão Sobrinho, Rodrigo Lacerda Nogueira, Daniel Salgado Kupper, Fabiana Cardoso Pereira Valera, Maurício Lacerda Nogueira, Luisa Lina Villa, Paula Rahal, Laura Sichero

**Affiliations:** 1 Laboratory of Genomic Studies, Universidade do Estado de São Paulo, UNESP, São José do Rio Preto, SP, Brazil; 2 Molecular Biology Laboratory, Center of Translational Oncology, Instituto do Câncer do Estado de São Paulo, ICESP, São Paulo, Brazil; 3 Department of Ophthalmology/Otorhinolaryngology and Head/Neck Surgery, Discipline Otorhinolaryngology, Faculty of Medicine of Ribeirão Preto, Universidade de São Paulo, USP, São Paulo, Brazil; 4 Laboratory of Research in Virology, Faculty of Medicine of São José do Rio Preto, FAMERP, São José do Rio Preto, Brazil; 5 Department of Radiology and Oncology, School of Medicine, Universidade de São Paulo, USP, São Paulo, Brazil; 6 School of Medicine, Santa Casa de São Paulo and HPV Institute, São Paulo, Brazil; University of Nebraska-Lincoln, UNITED STATES

## Abstract

A significant proportion of recurrent respiratory papillomatosis (RRP) is caused by human papillomavirus type 6 (HPV-6). The long control region (LCR) contains cis-elements for regulation of transcription. Our aim was to characterize LCR HPV-6 variants in RRP cases, compare promoter activity of these isolates and search for cellular transcription factors (TFs) that could explain the differences observed. The complete LCR from 13 RRP was analyzed. Transcriptional activity of 5 variants was compared using luciferase assays. Differences in putative TFs binding sites among variants were revealed using the TRANSFAC database. Chromatin immunoprecipation (CHIP) and luciferase assays were used to evaluate TF binding and impact upon transcription, respectively. Juvenile-onset RRP cases harbored exclusively HPV-6vc related variants, whereas among adult-onset cases HPV-6a variants were more prevalent. The HPV-6vc reference was more transcriptionally active than the HPV-6a reference. Active FOXA1, ELF1 and GATA1 binding sites overlap variable nucleotide positions among isolates and influenced LCR activity. Furthermore, our results support a crucial role for ELF1 on transcriptional downregulation. We identified TFs implicated in the regulation of HPV-6 early gene expression. Many of these factors are mutated in cancer or are putative cancer biomarkers, and must be further studied.

## Introduction

Recurrent Respiratory Papillomatosis (RRP) is characterized by the proliferation of multiple papillomas within the respiratory tract affecting especially the larynx [1,2]. RRP can affect persons of all ages; but a bimodal age distribution is often observed: disease peaks in children younger than 5 years and between the third and fourth decade of life. Thus, based on age distribution of affected individuals, RRP has been clinically divided into juvenile onset (JORRP) and adult onset RRP (AORRP) [3]. Human Papillomaviruses (HPV) types 6 and 11 are etiologically associated with RRP development [4]. Although RRP is considered a benign disease, high recurrence rates are associated to significant morbidity and is occasionally fatal [5].

The prototype HPV-6b clone was originally isolated from a condyloma acuminatum specimen [6]. Subsequently, HPV-6a and HPV-6vc non-prototypic genomes were identified by variations in restriction patterns, and gave rise to subtype classification [7]. Because the term subtype was redefined, HPV-6a, -6b, and -6vc were assigned to molecular variants since nucleotide sequence among these isolates diverges less than 2% within the *L1* gene [8]. Recently, the analysis of 190 complete viral genomes clustered HPV-6 isolates into two variants lineages named A and B with the latter enclosing 5 sublineages (differences in whole genome sequence of 0.5–1.0%) [9]. While the HPV-6b-reference isolate is one of the highly related members of lineage A, HPV-6vc and HPV-6a sort to B1 and B3 sublineages, respectively [10].

The viral long control region (LCR) comprises approximately 10% of the viral genome and encloses cis-regulatory elements for cellular and viral transcription factors (TFs) that modulate early gene expression and replication [11]. The repertoire of potential TFs binding sites within the LCR vary largely among distinct HPV types and variants [12,13] due to nucleotide divergences, and may impact binding affinity, transcriptional activity and ultimately the clinical outcome associated with HPV infections [14].

HPVs 16 and 18 intratype variability has been epidemiologically correlated to viral persistence and development of clinically relevant lesions [15–18]. Concerning the prevalence of low risk HPVs and the functional implications of viral heterogeneity, data is still scarce. In the present study, we thought to characterize the complete LCR of HPV-6 detected in a group of individuals diagnosed with RRP and analyze nucleotide variability and age of disease development. Further, we investigated the impact of LCR nucleotide heterogeneity upon transcription. In silico analysis revealed putative binding sites for GATA1, ELF1 and FOXA1 overlapping divergent nucleotide positions. We then analyzed the binding and influence of these TFs upon transcriptional activity of HPV-6 variants.

## Materials and Methods

### Clinical samples

We analyzed 23 laryngeal papillomas biopsy specimens from 13 patients diagnosed with RRP from 2005 to 2010, and treated at the Laryngology Clinic of the School of Medicine, University of São Paulo, Ribeirão Preto, Brazil. Disease severity was accessed using the Derkay system at each surgical intervention [19]. This study was approved by the Institutional Ethics Research Committee of the Sao Paulo State University at Sao José do Rio Preto, São Paulo, and written informed consent was obtained from patients or parents of underage patients.

### Sequence analysis

DNA was extracted using the QIAamp DNA Micro kit (Qiagen Inc.). The complete HPV-6 LCR (nt 7292 to 101) was amplified in two independent reactions. Amplicons were cloned using the pCR XL-TOPO Vector (Invitrogen), and clones were purified using the GeneJET Plasmid Miniprep Kit (Fermentas Life Sciences). Sequencing reactions were performed in an ABI 3130XL sequencer (Applied Biosystems) using the BigDye Terminator v3.1 Cycle Sequencing Kit (Applied Biosystems). Variants were identified by alignment of sequences with the references of HPV-6b (GenBank n° X00203), HPV-6a (GenBank n° L41216) and HPV-6vc (GenBank n° AF092932) using CLUSTALW [20] enclosed in the BioEdit 7.0.9.0 package [21]. Sequences were submitted to GenBank (accession numbers: KF436496-KF436509). TFs binding sites were predicted online (http://trap.molgen.mpg.de/cgi-bin/home.cgi) using the TRANSFAC database [22].

### Cell culture

C33A (ATCC HTB-31) and C33A cells permanently transfected with pGL3-HPV-6-LCR were grown in Dulbecco’s Modified Eagle medium supplemented with 10% fetal calf serum and antibiotics. Primary human foreskin keratinocytes (PHFK) (Clonetics), and PHFK infected with pLXSN-HPV-6b-*E6/E7* or pLXSN-HPV-16-*E6/E7* were maintained in keratinocyte serum free medium (KSFM), supplemented with 5ng/mL epidermal growth factor and 50μg/mL bovine pituitary extract (Invitrogen).

### Promoter activity assays

The complete LCR from the different HPV-6 variants was cloned upstream the luciferase gene in the pGL3-Basic vector (Promega). Accurate construction of all plasmids was confirmed by sequencing. Approximately 24 hours before transfection, 4x10^6^ C33A cells were plated in 10cm diameter dishes. Cells were co-transfected using Lipofectamine (Invitrogen) with 4μg of pGL3-LCR-Luc plasmids and 1μg of pCMV-βGal vector for internal control of transfection efficiency [23]. Extracts were obtained 48 hours after transfection by addition of 800μL of 1X Reporter Lysis Buffer (Promega). Luciferase activity was measured using the Promega Luciferase Assay System kit (Promega) in a Victor Light Luminescence Counter (Perkin Elmer). The β-Galactosidase Enzyme Assay System (Promega) was used to determine β-Galactosidase activity at 420nm on a Benchmark plate reader (Bio-Rad). Relative luciferase activity measurements were normalized to β-Galactosidase activity and protein content measured using the Bradford assay (Bio-Rad). Averages were based on the mean of triplicates of three independent experiments.

### Validation Assays

C33A cells were co-transfected in 96-well plates using Fugene (Promega) with 100ng of pGL3-LCR-Luc plasmids of different variants of HPV-6 together with increasing amounts of selected TFs expression plasmids (120, 240, 360ng), and 50ng of pCMV-β-Gal vector. Cells were harvested 48 hours after transfection by addition of 100μL of 1X Reporter Lysis Buffer (Promega). Relative luciferase activity was normalized to β-galactosidase measurements. Transcriptional activity was compared to basal activity of the same variant with no TFs superexpressed. Averages and standard deviations are based on the mean of triplicates of at least twelve independent experiments.

### Statistical Analysis

Fisher´s exact test was used to compare HPV-6 variant distribution between JORRP and AORRP cases. We used T-test to compare Derkay mean values with 95% confidence interval (CI) between HPV-6a and -6vc-related RRP cases. These analyses were conducted using the GraphPad Prism statistical package (version 5.01). Kruskal-Wallis and Tukey’s tests were employed to compare LCR activity among HPV-6 variants. For TFs validation assays, one-way analysis of variance and Tukey’s multiple comparison tests were performed using the SPSS statistical package version 20.0. P< 0.05 was considered significant for all tests.

### Chromatin Immunoprecipitation (ChIP)

ChIP assays were perfomed using the MAGnify Chromatin Immunoprecipitation System (Invitrogen). Briefly, C33A cells permanently transfected with pGL3-HPV-6-LCR from different variants were cross-linked with methanol-free formaldehyde (Polyscience) and further sonicated using a Sonic Dismembrator Model 100 (Fisher Scientific). Production of DNA fragments of 100-500bp was checked by gel electrophoresis in agarose 2%. Approximately 1x10^5^ cells were incubated with 20μg of anti-GATA1 (Abcam), anti-ELF1 (Santa Cruz Biotechnology), or anti-FOXA1 (Santa Cruz Biotechnology) overnight at 4°C with moderate agitation. Recovered DNA was subjected to PCR using specific primers surrounding putative TFs binding sites.

### Western blotting

Cells were washed with ice-cold phosphate-buffered saline (PBS), scraped, and centrifuged. Radio immunoprecipitation assay (RIPA) buffer (20mM Tris-HCl [pH 7.4], 150mM sodium chloride, 1mM ethylenediaminetetraacetic acid, 1mM ethylene glycol tetraacetic acid, 0.1% sodium dodecyl sulfate [SDS], 1% sodium deoxycholate, 1% Triton X-100, 1mM sodium orthovanadate) containing complete protease inhibitor cocktail (Roche) was used for protein extraction. Eighty micrograms of each lysate were submitted to 12% SDS polyacrylamide gel electrophoresis, and further transferred to nylon membranes (Hybond). Incubations with anti-GATA1 (1:500), anti-ELF1 (1:200), anti-FOXA1 (1:500), or anti-tubulin (1:1.000) (Sigma) were conducted overnight in PBS-T (PBS, 0.05% Tween 20) at 4°C with 5% milk. We followed incubation with anti-HRP-conjugated antirabbit secondary antibody (1:5.000) (GE Healthcare) for 1 hour at room temperature. Proteins were visualized using the Amersham ECL Western Blotting Detection Reagents (GE Healthcare) in a ImageQuant LAS4.000 equipment (GE Healthcare), and quantified using the ImageQuant TL software (GE Healthcare).

## Results

### Human papillomavirus (HPV) type 6 variant characterization and distribution

We sequenced the LCR of 23 HPV-6 RRP specimens obtained from 13 patients. Individuals with more than one biopsy harbored identical HPV genomes. We detected 7 different HPV-6 variants (Table 1).

**Table 1:** Nucleotide sequence variability within the long control region (LCR) of human papillomavirus type 6 (HPV-6) molecular variants.

Number of samples	HPV-6 LCRvariant	7320	7350	7520	7626	7631	7633	7681	7762	7875	7919	7978	16
2	6a-ref	A	G	C	T	A	A	A	C	C	A	C	G
1	6a-var1	·	·	·	·	·	·	·	·	·	·	·	A
1	6a-var2	G	·	·	·	·	·	·	·	·	·	·	·
6	6vc-ref	·	T	I1	·	T	I2	·	G	A	C	·	·
1	6vc-var1	·	T	I1	G	T	I2	·	G	A	C	·	·
1	6vc-var2	·	T	I1	·	T	I2	·	G	A	C	T	·
1	6vc-var3	·	T	I1	·	T	I2	G	G	A	C	·	·

Genomic positions containing specific mutations are indicated vertically across the top. Genomic positions without mutations compared to the HPV-6a-ref sequence (·), Insertions (I): I1 = TTATTGTATATCTTGTTACA; I2 = C nucleotide insertion.

Maximum genomic distance was 7 mutations between HPV-6a-ref and -6vc-ref (2 insertions and 5 substitutions). HPV-6a-var1, -6a-var2, -6vc-var1, -6vc-var2 and -6vc-var3 have unique substitutions at nucleotide positions 16 (G→A), 7320 (A→G), 7626 (T→G), 7978 (C→T) and 7681 (A→G), respectively. We detected HPV-6a-related sequences (HPV-6a-ref,-var1,-var2) in 32.5% (4/13) patients. Additionally, 67.5% (9/13) individuals contained HPV-6vc-related sequences (HPV-6vc-ref,-var1,-var2,-var3), of which HPV-6vc-ref was the most prevalent.

Age at RRP diagnosis ranged from newborn to 51 years old. All JORRP cases harbored HPV-6vc-related variants, whereas most AORRP specimens had HPV-6a-related variants (66.6%; 4/6) (p = 0.02) (Table 2). Average Derkay scores were similarly distributed in patients with different variants.

**Table 2:** Clinical data of recurrent respiratory papillomatosis (RRP) patients harboring human papillomavirus type -6a and -6vc (HPV-6a and -6vc) related variants.

	HPV-6a-related	HPV-6vc-related	p-value
JORRP	n = 0	n = 7	P = 0.02^a^
AORRP	n = 4	n = 2	
Mean Derkay score	6.4 (CI 95%: 4.8 to 7.9)	6.8 (CI 95%: 6.3 to 7.3)	P = 0.46^b^

Abbreviations: JORRP, juvenile onset recurrent respiratory papillomatosis; AORRP adult onset recurrent respiratory papillomatosis.

^a^ Fisher's exact test.

^b^ T-test.

### Transcriptional activity of human pappilomavirus (HPV) type 6 variants

**Fig 1:**
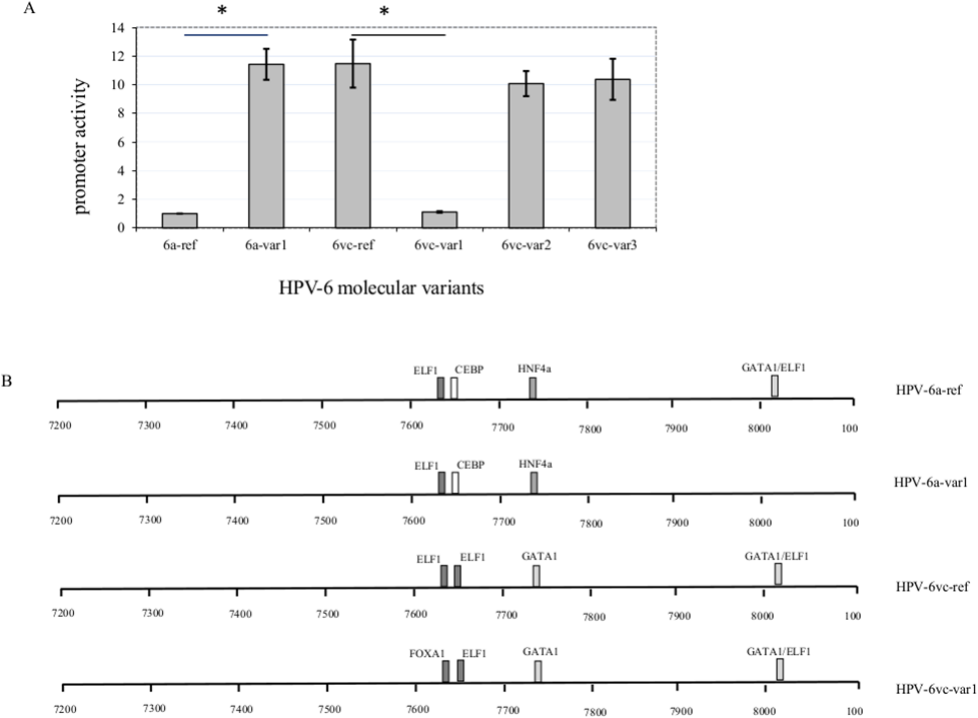
Molecular variants of HPV-6 differ in early promoter activity. (A) Transcriptional activity of human papillomavirus type (HPV-6) molecular variants isolated from recurrent respiratory papillomatosis (RRP) cases. Data are presented as mean ± SD relative values from 3 independent experiments. Transcriptional activity was normalized to that of HPV-6a-ref which was arbitrarily defined as the reference and set to value 1. Kruskal-Wallis and Tukey’s tests were used to compare LCR activity among the different HPV-6 molecular variants. Asterisks indicate isolates that showed statistically significant different transcriptional activities compared to the corresponding references (HPV-6a-ref or HPV-6vc-ref). (P<0.001). (B) Putative cellular transcription factors binding sites within the long control region (LCR) that differ among the different molecular variants of human papillomavirus (HPV) type 6 detected.

Luciferase assays were used to access the impact of LCR nucleotide sequence heterogeneity on early viral transcription. Unfortunately, we were unable to construct a recombinant plasmid containing the HPV-6a-var2 LCR. We observed an 11.5 fold enhanced promoter activity for HPV-6vc-ref variant as compared to HPV-6a-ref. A transition at nucleotide position 16 detected solely in HPV-6a-var1 led to an increase in transcriptional activity similar to that of the HPV-6vc-ref, whereas the transversion at position 7630 inherent of the HPV-6vc-var1 abolished the increased activity observed for HPV-6-vc-ref. HPV-6vc-var2 and -6vc-var3 showed promoter activities similar to HPV-6vc-ref (Fig 1A)

### Putative cis-elements within HPV-6 LCR

Search for putative TFs binding sites was restricted to nucleotide positions divergent among HPV-6a-ref, -6a-var1, -6vc-ref, -6vc-var1 because these isolates showed differences in transcription. This approach revealed that nucleotide heterogeneity of HPV-6 variants may impact on binding of different TFs including FOXA1, GATA1 and ELF1 (Fig 1B).

### FOXA1, ELF1 and GATA1 binding and influence upon HPV-6 LCR

**Fig 2:**
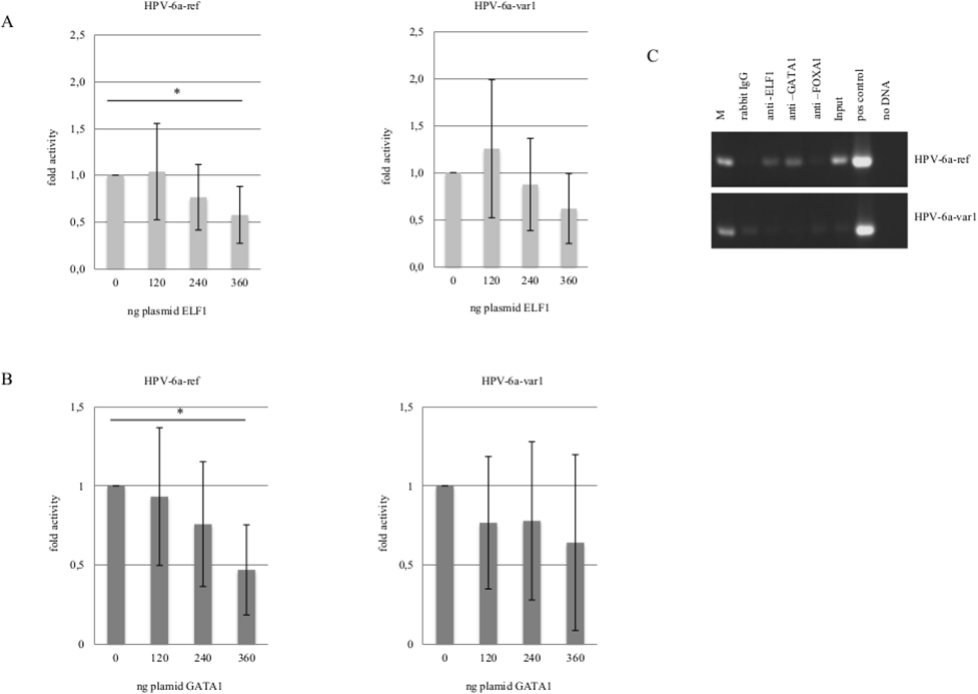
Binding and activity of ELF1 and GATA1 to HPV-6a-ref and HPV-6a-var1. Luciferase reporter assay for (A) ELF1 and (B) GATA1. (C) Amplification of LCR fragments following chromatin immunoprecipitation (ChIP) using primers surrounding nucleotide position 16 that differs among HPV-6a-ref and HPV-6a-var1 variants. Input-nonimmunoprecipated samples.

The substitution in nucleotide position 16 (G→A) detected in HPV-6a-var1 leads to the elimination of putative binding sites for GATA1 and ELF1 as compared to the HPV-6a-ref (Fig 1B). We observed by transfecting increasing amounts of plasmids expressing GATA1 or ELF1 that both TFs significantly reduce HPV-6a-ref transcription, whereas have no significant impact upon HPV-6a-var1 LCR (Fig 2A). ChIP assays were performed to verify the functional activity of the cis-elements suggested in silico. PCR amplification of recovered DNA using primers surrounding nucleotide position 16 revealed the in vivo association of both ELF1 and GATA1 with the HPV-6a-ref LCR but not with the HPV-6a-var1 isolate suggesting a direct effect upon transcriptional activity (Fig 2B).

**Fig 3:**
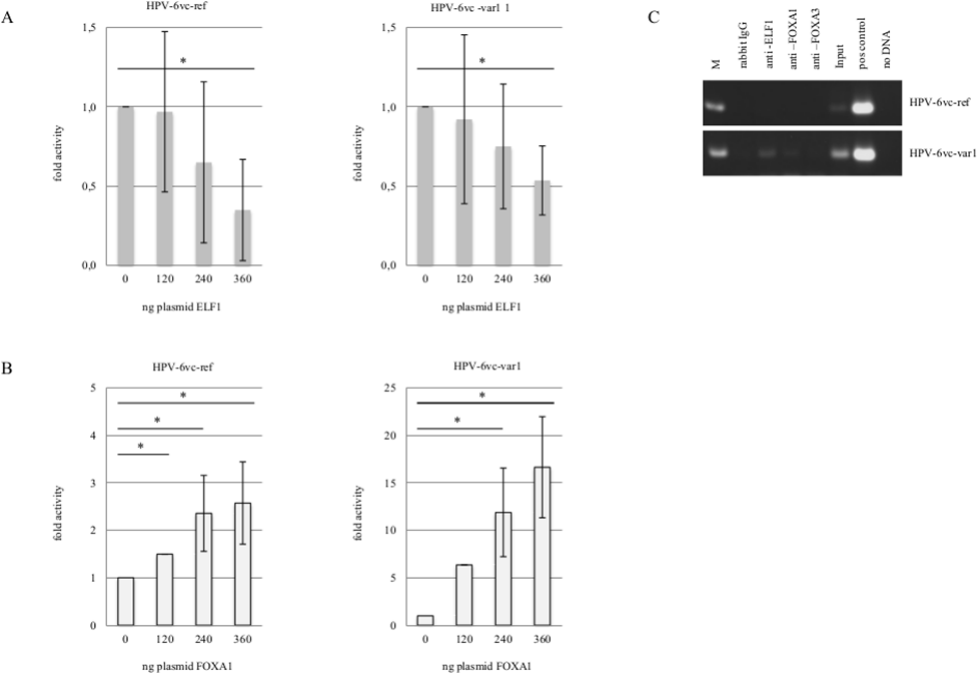
Binding and activity of ELF1 and FOXA1 to HPV-6vc-ref and HPV-6vc-var1. Luciferase reporter assay for (A) ELF1 and (B) FOXA1. (C) Amplification of LCR fragments following chromatin immunoprecipitation (ChIP) using primers surrounding nucleotide position 7626 that differs among HPV-vc-ref and HPV-6vc-var1 variants. Input-nonimmunoprecipated samples.

Divergence in nucleotide position 7626 (T→G) between HPV-6vc-ref and HPV-6vc-var1 results in the replacement of a putative ELF1 for a FOXA1 binding site as suggested by computational analysis (Fig 1B). ELF1 inhibited transcription activity of both isolates in a similar manner (Fig 2A). Although FOXA1 significantly transactivated HPV-6vc-ref, enhancement of transcription was much stronger for the HPV-6vc-var1 isolate. Interestingly, both FOXA1 and ELF1 were shown to bind solely to the HPV-6vc-var1 LCR; no binding of both protein to the HPV-6vc-ref was detected by ChIP (Fig 3B).

**Fig 4:**
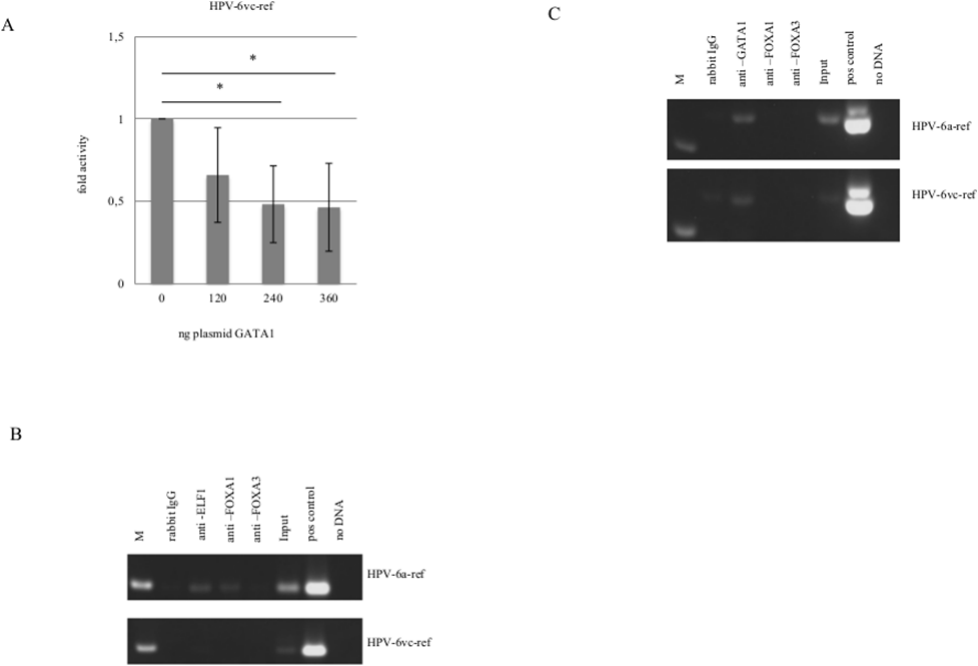
Binding and activity of ELF1, GATA1 and FOXA1 to HPV-6a-ref and HPV-6vc-ref. (A) Luciferase reporter assay for GATA1. Amplification of LCR fragments following chromatin immunoprecipitation (ChIP) using primers surrounding nucleotide position (B) 7631/33 and (C) 7762 and that differ between HPV-6a-ref and HPV-6vc-var1 variants. Input-nonimmunoprecipated samples.

Nucleotide differences in positions 7631/33 leads to a substitution of a putative CEBP for an ELF1 binding site in HPV-6vc-ref as compared to HPV-6a-ref (Fig 1B). Additionally, alteration in nucleotide position 7762 determines the replacement of a putative HNF4a for a GATA1 binding site in HPV-6vc-ref as compared to HPV-6a-ref. ELF1 significantly repressed both HPV-6a-ref and HPV-6vc-ref (Figs 2A and 3A). However, ELF1 binding to HPV-6vc-ref LCR was not observed. In this case, ELF1 is most probably bound to the the ELF1 binding site at nucleotide position 7626 within the HPV-6a-ref LCR. Furthermore, we observed that GATA1 represses transcription of both HPV-6a-ref and HPV-6vc-ref (Figs 2A and 4A), although repression was more pronounced for the HPV-6vc-ref. GATA1 was shown to bind the LCR of both variants using ChIP (Fig 4B). We observed that ELF1, GATA1 and FOXA1 are detected not only in PHFK, but also in keratinocytes transducing *E6/E7* from both HPV-16 and HPV-6b (S1 Fig).

## Discussion

RRP is considered a non-malignant disease; however, in more severe clinical cases lesions spread throughout the lower respiratory tract, affecting bronchi, trachea, esophagus and lung [24]. HPV-6 and -11 DNA are detected in more than 90% of the cases [4]. While HPV-6 is more prevalent in RRP individuals, HPV-11 infection has been linked to a more aggressive clinical outcome [25]. Besides, co-infection with different variants of HPV-6 and -11 is very rarely detected [25, 26] and it has been suggested that RRP is the consequence of persistent infection with the initial viral genomic variant [27]. HPV-6 variants distribution is not as geographically restricted as has been reported for HPV-16 and -18 variants [9,28]. However, a possible lesion-specific preference of some HPV-6 variants was suggested due to the association of HPV-6vc-related isolates with anogenital infections [9].

We evaluated the complete HPV-6 LCR sequence of 13 laryngeal biopsies from RRP patients, and describe for the first time 5 novel HPV-6 LCR variants: HPV-6a-var1, -6a-var2, -6vc-var1, -6vc-var2 and -6vc-var3. In this series, we observed uneven distribution of HPV-6 variants: HPV-6vc and HPV-6a-related variants were more prevalent in JORRP and AORRP cases, respectively. It has been reported that pregnant women with evident anogenital warts or recent HPV infection at the time of delivery are more prone of having children affected by JORRP. Thus, the predominance of HPV-6vc-related variants in JORRP in this series could reflect the predominance of these genotypes in anogenital warts and anal cancer samples, as previously described [9,29,30]. HPV-6vc-related variants were also frequently detected in laryngeal papillomas from Australia [29], Slovenia [31] and South Africa [32]. However, comparison among studies is hindered by the lack of clinical information and restriction of analyses to pediatric cases in these reports. Even though some severe RRP cases are observed in adults, JORRP is a more aggressive condition being associated with increased risk of lesion development in the lower respiratory tract [33]. Furthermore, there is a relation of age and surgical procedures, and in the case of JORRP, due to the small airway of children, multiple surgeries are required to avoid airway obstruction [33]. Although the regularity and number of surgical interventions in an individual is considered when estimating Derkay values, average scores were not differently distributed in individuals harboring different molecular variants of HPV-6 in the series of cases we analyzed. The lack of clinical correlates of disease requires the study of larger series. Nevertheless, it is of note that in addition to age, other host factors such as genetic and immunological profiles appear to contribute to the greater aggressiveness of RRP [34,35].

LCR activity is crucial for viral replication, transcription and host cell proliferation mediated by these viruses [11]. Some studies described the impact of high-risk HPV-16 and -18 LCR variability upon viral early transcriptional activity, which may support augmented E6 and E7 levels and finally confer enhanced oncogenic potential to specific variants [14,36,37]. Furthermore, LCR heterogeneity inherent of HPV-16 variants has been shown to influence expression of viral E2 and E1 replicating proteins and affect viral replication efficiency [38]. Concerning low risk viral types, a duplication of the HPV-11 early viral promoter sequence was associated to a higher degree of disease severity [39]. Additionally, increased LCR activity of HPV-11 variants was shown to associate with clinically RRP aggressiveness (30 to 33 episodes) [26]. Thus, one may hypothesize that infection with more replicative HPV variants could result in more efficient proliferation of papillomas and thus respond for the augmented frequency of surgical processes required to control disease and avoid respiratory hitch.

Some differences were observed in transcriptional activity among the HPV-6 molecular variants analyzed. In silico analysis revealed that some nucleotide position in which substitutions were detected overlap putative TF binding sites. Further, cis-acting elements were created or abolished due to nucleotide heterogeneity. Even so, we may not discard the indirect influence of some cellular TFs upon HPV transcription. The substitution in nucleotide position 16 of HPV-6a-var1 isolate in the background of the HPV-6a-ref sequence eliminates putative GATA1 and ELF1 binding sites. GATA1 is one of the members of GATA TFs family and has been linked to cancer development associated to chromosome 21 trisomy [40]. Further, GATA1 is a master regulator of erythroid cell development [41]. ELF1 is mainly expressed in lymphoid cells and is essential for the regulation of hematopoiesis and angiogenesis during development [42]. Once CHIP assays revealed the in vivo association of both TFs exclusively to the HPV-6a-var1 LCR, we believe that GATA1 and ELF1 induced inhibition of HPV-6a-ref transcription is determinant for the diminished transcriptional activity of this isolate as compared to the HPV-6a-var1.

HPV-6vc-var1 LCR was approximately 11 times less active than the HPV-6vc-ref. The single nucleotide alteration that differentiates these LCR leads to loss and creation of a putative ELF1 and FOXA1, respectively. FOXA1 is a pioneer factor which binding to promoters and enhancers sequences permits the access of other specific TFs to the chromatin [43]. FOXA1 has recently been shown by us to strongly enhance HPV-16 and -18 transcriptions [14]. Among the TFs tested by us, FOXA1 was the only protein that enhanced HPV-6 transcription. Even though FOXA1 was shown to activate much more HPV-6vc-var1 as compared to HPV-6vc-ref, and ELF1 repressed both variants transcriptional activity similarly, the binding of ELF1 solely to the HPV-6vc-var1 may explain the reduced transcriptional activity observed for this isolate. Particular changes inherent of HPV-6vc-var2 and -6vc-var3 did not influence the LCR activity. Although computational analysis indicated a putative GATA1 binding site exclusively in HPV-6a-ref as compared to HPV-6vc-ref, we observed that this TF was able to interact in vivo and decrease transcriptional activity of both variants. For this reason, we hypothesize that ELF1 binding and repression of viral transcription is crucial for the lower transcriptional activity inherent of HPV-6a-ref.

Altogether, we described five novel HPV-6 LCR variants. We report uneven distribution of HPV-6 variants in JORRP and AORRP; HPV-6vc-related variants were the only isolates detected among JORRP cases. Overall, HPV-6vc-related variants were the more transcriptionally active, and we further identified cellular TFs capable of influencing transcriptional activity of the different HPV-6 isolates. Our results support a crucial role of ELF1 on transcriptional downregulation in the differences observed. Further studies might shed light on these findings.

## Supporting Information

S1 Fig Transcription factors levels in primary human foreskin keratinocytes. (A) ELF1, (B) GATA1 and (C) ELF1 levels in primary human foreskin keratinocytes (PHFK), and PHFK infected with pLXSN-HPV-6b-E6/E7 or pLXSN-HPV-16-E6/E7. One representative experiment from two is shown.
(TIFF)
