# Supplementary figures and images for: Differences in Transcriptional Activity of Human Papillomavirus Type 6 Molecular Variants in Recurrent Respiratory Papillomatosis
Source: PLoS One. 2015 Jul 7;10(7):e0132325. doi: 10.1371/journal.pone.0132325 (PMC4494706; doi:10.1371/journal.pone.0132325)

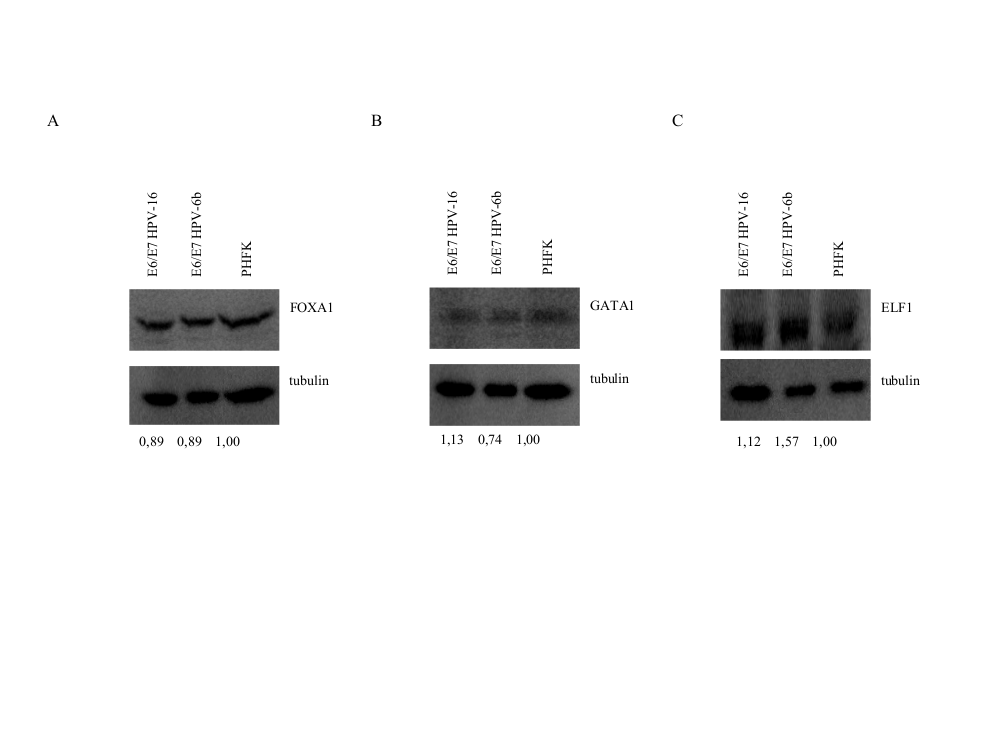

Supplement: S1 Fig — (A) ELF1, (B) GATA1 and (C) ELF1 levels in primary human foreskin keratinocytes (PHFK), and PHFK infected with pLXSN-HPV-6b-E6/E7 or pLXSN-HPV-16-E6/E7. One representative experiment from two is shown. (TIFF) [file pone.0132325.s001.tiff]
